# Effectiveness of physical education interventions on physical and mental health in pre- and school-aged children: a systematic review and meta-analysis

**DOI:** 10.3389/fpubh.2026.1757982

**Published:** 2026-04-30

**Authors:** Qinglei Wang, Nina Wang, Mengyan Wang

**Affiliations:** 1 School of Physical Education, Guangdong University of Petrochemical Technology, Maoming, Guangdong, China; 2 Faculty of Liberal Arts and Law, Guangdong University of Petrochemical Technology, Maoming, Guangdong, Malaysia; 3 Faculty of Education, Universiti Malaya, Kuala Lumpur, Malaysia

**Keywords:** mental health, meta-analysis, physical education, pre-school children, school-aged children

## Abstract

**Introduction:**

This systematic review and meta-analysis aimed to evaluate the efficacy of school-based physical education interventions for improving pre- and school-aged children’s physical and mental health outcomes (3–12 years) and to examine whether effects vary across intervention and outcome types.

**Method:**

Following PRISMA 2020 guidelines, an extensive search was conducted in Ovid MEDLINE, Embase, the Cochrane Controlled Trials Registry, and Scopus up to October 2023. Randomized controlled trials evaluating PE- or school-day activity–based interventions targeting physical and/or mental health outcomes in preschool (3–6 years) and school-aged children (6–12 years) were included. Random-effects meta-analysis (REML) was performed using standardized mean differences (SMDs), and heterogeneity was assessed using Q and I^2^ statistics. Subgroup analyses and influence diagnostics were conducted to explore between-study variability”.

**Results:**

Nineteen studies were included in the analysis. The results of the meta-analysis revealed no significant evidence of the effectiveness of these interventions on physical or mental health (1.61, 95CI; −1.37, 4.60; *p* = 0.29). The subgroup analysis results showed no significant difference between physical activity (0.05, 95%CI: −0.20, 0.29) and psychological exercise (0.06, 95%CI: −0.36, 0.24) (*p* = 0.58). Notably, substantial heterogeneity across studies indicates variability in the interventions’ effects.

**Conclusion:**

The evidence remains inconclusive due to substantial heterogeneity, variability in intervention content, and differences in outcome measurement. Future trials should adopt standardized outcome measures, improve reporting of intervention implementation, and strengthen methodological rigor to enable more definitive synthesis of physical education intervention effects in children.

## Introduction

The pivotal role of physical education in nurturing the holistic health of pre- and school-aged children stands as an established cornerstone in child development. Beyond the mere acquisition of motor skills, physical education serves as a conduit for fostering an intrinsic understanding of the significance of an active lifestyle, instilling values that transcend the confines of a classroom or playground. Extensive research amplifies the multifaceted advantages inherent in physical education, painting a comprehensive picture that delineates its profound impact, not only on physical well-being but also on cognitive acuity and emotional resilience, thus positioning it as an indispensable cornerstone for the holistic development of young learners (1).

The effect of physical education on mental health outcomes is of paramount importance. Integrating physical activity with physical education has effectively alleviated depression and anxiety symptoms among non-clinical populations (1). Rebar et al.’s (1) in meta-analysis found positive impacts on depression and anxiety symptoms, suggesting daily physical education promotes mental health improvement.

Physical education programs can greatly benefit children’s well-being, as evidenced by Ahn and Fedewa’s (2) meta-analysis revealing a significant positive correlation between physical activity and mental health across all body weights. Their study suggests integrating physical education interventions in schools would promote holistic well-being for all children, regardless of physical attributes (2).

Physical education positively impacts emotional competence beyond reducing distress, according to Cho’s (3) meta-analysis. The study found physical education (PE) interventions effectively foster students’ emotional proficiency abilities (2, 3). Cooperative learning interventions in physical education significantly enhance students’ intrinsic motivation, increasing interest and enjoyment in physical activities, thereby positively impacting overall mental well-being (4).

Physical education benefits mental health across the lifespan. Perry et al.’s (5) meta-analysis found parental mental illness challenges children’s physical well-being, underscoring the need to address parental mental health to improve offspring’s physical health. Despite extensive evidence highlighting the multifaceted benefits of physical education interventions on holistic child development, a critical gap remains in comprehensively synthesizing their effectiveness specifically for preschool and school-aged populations. While physical education programs have demonstrated positive impacts across physical, cognitive, emotional, and social domains, potential drawbacks or challenges in their real-world implementation and sustained engagement for these key developmental stages warrant further investigation.

Recognizing this need, we aim to conduct a systematic review and meta-analysis focusing on preschool and school-based children. This analysis seeks to evaluate the effectiveness of physical education interventions in enhancing the physical and mental health of pre- and school-aged children, while also identifying potential barriers or facilitators to successful program delivery and participation during this crucial period.

## Methods

### Source of data

The systematic review adhered to the PRISMA 2020 guidelines (6). A meticulous and comprehensive search strategy, employing relevant keywords, was executed across multiple databases, including Ovid MEDLINE, Embase, Cochrane Controlled Trials Registry, and Scopus, to identify pertinent studies. A completed PRISMA checklist indicating where each item is addressed is provided in PRISMA 2020 checklist. The literature search was conducted up to October 2023.

### PRISMA 2020 checklist

A completed PRISMA 2020 checklist that maps each reporting item to the corresponding page/section in this manuscript is provided.

### Inclusion criteria

To be eligible for study, the population should consist of pre- and school-aged children, approximately between 3 and 12 years old, encompassing all genders. The intervention should involve physical education interventions for both physical and mental health. Specifically, children with overweight/obesity were required to be free from any medical conditions, and the study needed to be a randomized controlled trial (RCT). The outcomes measured are physical health [such as fitness and body mass composition (BMI)] and mental health (including psychological well-being and cognitive functioning). It’s important to note that our selection criteria were limited to trials published in the English language. The two reviewers autonomously performed the searches and conducted the initial phase of study selection, involving the examination of titles and abstracts. In the subsequent stage, full-text studies were assessed for inclusion based on eligibility criteria. Oversight of the process and resolution of any discrepancies were overseen by a third author. Potential studies were also sought in the reference lists of eligible publications and comparable systematic reviews. The management of references was facilitated using EndNoteX20 (Malaysia, Kuala Lumpur, UM Library-PTM) provided by Thomson Reuters.

### Extraction of data

Two reviewers independently screened titles/abstracts and then full texts against the eligibility criteria. Discrepancies were resolved by discussion, with third-reviewer arbitration when required. Two reviewers independently extracted data using a standardized form (study design, sample characteristics, intervention content/dose, comparator, outcome measures, time points, and statistics required for effect size calculation). When multiple reports described the same trial, data were collated and counted once.

### Quality appraisal of included studies

Risk of bias was assessed independently by two reviewers using the Cochrane Risk of Bias 2 (RoB 2) tool for randomized trials, with disagreements resolved by discussion (or third reviewer).

### Synthesis of data

In this project, we calculated pooled effect sizes and conducted an overall evaluation of the relationship between interventions and health outcomes. Additionally, we performed subgroup analyses to identify potential sources of heterogeneity, such as participant characteristics and unique intervention attributes.

### Statistical analysis

A random-effects model was employed for statistical analysis to model the data effectively. The restricted maximum-likelihood estimator was adopted to quantify the extent of heterogeneity (7). The evaluation of heterogeneity included the application of the Q-test, an approach introduced by Cochran (8), along with the I^2^ statistic (8–10).

To identify any outliers or influential factors in the analytical model, studentized residuals and Cook’s distances were examined. Any studies with studentized residuals above the threshold of the 100 × (1–0.05/(2 × k)) th percentile of a standard normal distribution were considered potential outliers, using a Bonferroni correction with a two-sided *α* = 0.05, taking into account the total of k studies in the meta-analysis. Furthermore, studies with Cook’s distances exceeding the median plus six times the interquartile range of Cook’s distances were classified as influential (11).

All analytical processes were executed using R software (version 4.3.0) in conjunction with the metafor (7, 12).

### Publication bias

To evaluate the presence of funnel plot asymmetry, the rank correlation test employed established by Begg and Mazumdar (13) and the regression test developed by Sterne and Egger were employed (14).

### Strength of evidence

The strength of the evidence was evaluated using the GRADE (Grading of Recommendations, Assessment, Development, and Evaluation) approach. This approach considers factors such as the study design, risk of bias, consistency of results, estimates’ precision, and evidence’s directness.

## Results

### Study characteristics

**Figure 1:**
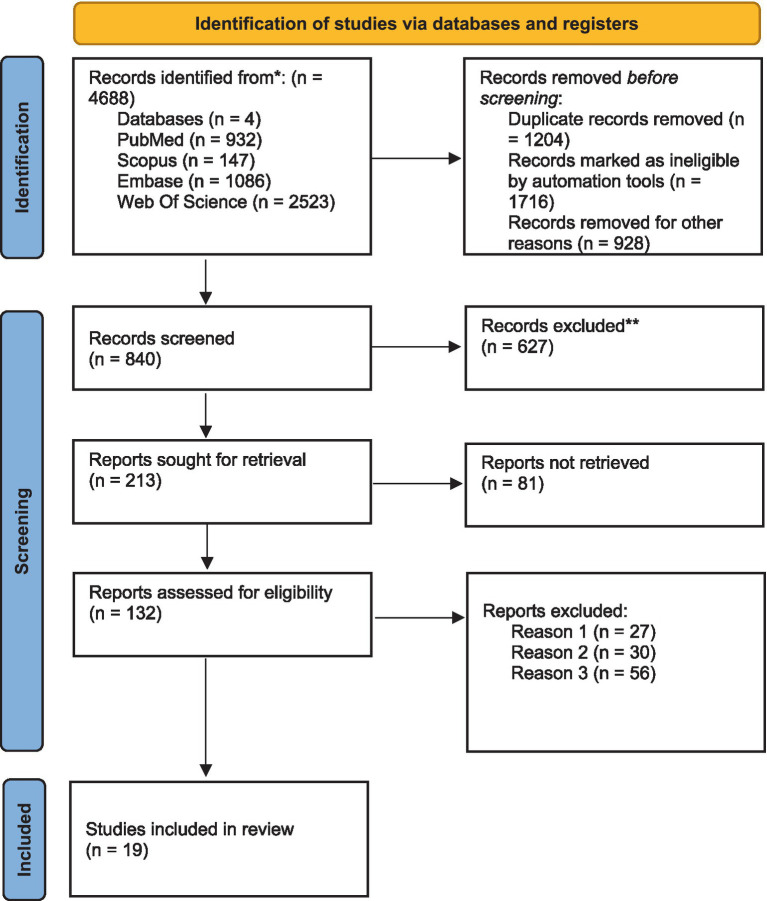
PRISMA flow diagram.

This comprehensive review encompassed an analysis of 19 studies of 4,688 studies involving a total of 4,719 participants. Detailed information regarding screening process flow can be found in Figure 1 of the PRISMA flowchart. These individuals were divided into two groups: the intervention group consisting of 2,209 people, and the control group with 2,510 people. The studies evaluated interventions in both physical education and psychological domains, lasting between 3 and 144 weeks with an average duration of 73.5 weeks. The studies measured outcomes related to active math and language, active lessons integrated into various curriculum, yoga, physical exercise, and football. Additional details for each individual RCT can be found in Table 1.

**Table 1:** Characteristics and outcomes of research studies.

Author	Participants	Intervention	Duration (week)	Outcome	Risk of bias
De Greef et al. (2015) (20), Netherlands	499, INT (*n*: 249), CON (*n*: 250), age: 8.1	Active Maths and Language	22	Cardiovascular fitness	Low
De Greef et al. (2015) (20), Netherlands	499, INT (*n*: 249), CON (*n*: 250), age: 8.1	Active Maths and Language	22	Muscular fitness	Low
De Greef et al. (2016) (21), Netherlands	376, INT (*n*: 181), CON (*n*: 195), age: 8.1	Active Maths and Language	22	BMI, Muscular fitness, Cardiovascular fitness	High
Donnelly et al. (22), Australia	24, INT (*n*: 14), CON (*n*: 10), age: 7.8	Active lessons across curriculum	144	BMI	Low
Donnelly et al. (2009) (22), USA	584, INT (*n*: 316), CON (*n*: 268), age: 8.1	Active lessons across curriculum	144	Muscular fitness	High
Have et al. (2018) (23), Denmark	505, INT (*n*: 294) (*n*: 211), age: 7.2	Active Maths	48	BMI	Low
Liu et al. (2008) (24) and Have et al. (2018) (23), China	753, INT (*n*: 328), CON (*n*: 425), age: 9.2	Active lessons across curriculum	36	BMI	High
Vetter et al. (2018) (25), Australia	88, INT (*n*: 44), CON (*n*: 44), age: 9.8	Active Maths	6	BMI	High
Beck et al. (2016) (15), Denmark	165, INT (*n*: 57), CON (*n*: 108), age:7.5	Gross Motor Math	6	Learning and intelligence	Low
Callcott et al. (2014) (26), Australia	297, INT (*n*: 100), CON (*n*: 197), age: 4.5	Active literacy teaching	48	Learning and intelligence	High
Elofsson et al. (2016) (27), Sweden	53, INT (*n*: 26), CON (*n*: 27), age: 5.8	Active Maths	3	Learning and intelligence	High
Fedewa et al. (2015) (28), USA	460, INT (*n*: 156), CON (*n*: 304), age: 9.8	Active lessons across curriculum	32	Learning and intelligent	Low
Graham et al. (2014) (29), USA	21, INT (*n*: 10), CON (*n*: 11), age: 7.3	Active Maths	1 day	Learning and intelligence	High
Kirk and Kirk (2013) (30), USA	72, INT (*n*: 36), CON (*n*: 36), age: 3.8	Active literacy lessons	24	Learning and intelligence	Low
Mavilid et al. (2016) (31), Australia	87, INT (*n*: 27), CON (*n*: 29), age: 4.9	Active Geography lesson	1 day	Learning and intelligence	Low
Telles et al. (2013) (32), India	98, INT (*n*: 49), CON (*n*: 49), age: 10.4	Yoga and physical exercise	12	BMI	Medium
Seabra et al. (2014) (33), USA	20, INT (*n*: 12), CON (*n*: 8), ages: 10.3	Football	20	BMI	Low
Telles et al. (2013) (34), India	98, INT (*n*: 49), CON (*n*: 49), age: 104	Yoga and physical exercise	12	Total Score of self-esteem	Medium
Seabra et al. (2014) (33), USA	20, INT (*n*: 12), CON (*n*: 8), age: 10.3	Football	20	Total Score of self-esteem	Low

INT: intervention group; CON: control group.

### Assessment of risk of bias

The Cochrane risk-of-bias tool was used to evaluate the robustness of the included studies. Applying the GRADE criteria, the overall level of certainty in the evidence ranged from low to moderate. Considerations of the risk of bias and potential inconsistencies within the included studies primarily influenced this grading.

### Meta-analysis

**Figure 2:**
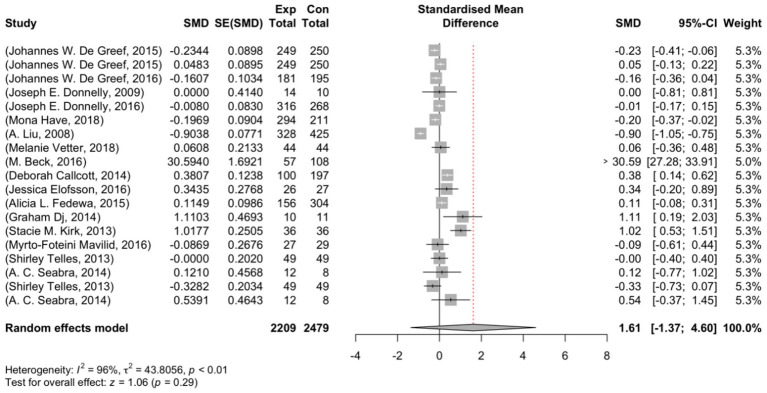
Forest plot showing the observed outcomes and the estimate of the random-effects model.

The range of observed outcomes standardized mean difference (SMD) was vast, varying from −0.904 to 30.594. The majority of estimates showed positive effects, accounting for 53%. A random-effects model obtained an estimated average effect size (*μ*) of 1.614 (95% CI: −1.369–4.597). Although the average appears positive, the statistical analysis revealed that the effect size average did not differ significantly from zero (z = 1.061, *p* = 0.289). Figure 2 visually displays this information through a forest plot that depicts the observed outcomes and the estimate from the random-effects model. Additionally, a Q-test investigation showed significant heterogeneity among the real outcomes (Q (18) = 497.267, *p* < 0.001, τ^2^ = 43.806, I^2^ = 99.958%). It is important to note that there is variability in the outcomes of studies. Although the overall effect seems to be positive, we must acknowledge the existence of studies that may have negative outcomes.

After examining the studentized residuals, it was discovered that one study conducted by Beck et al. (15) had a value exceeding ± 3.008, which could indicate a potential outlier status in the model. Additionally, based on Cook’s distance, the same study by Beck et al. (15) was identified as influential.

**Figure 3:**
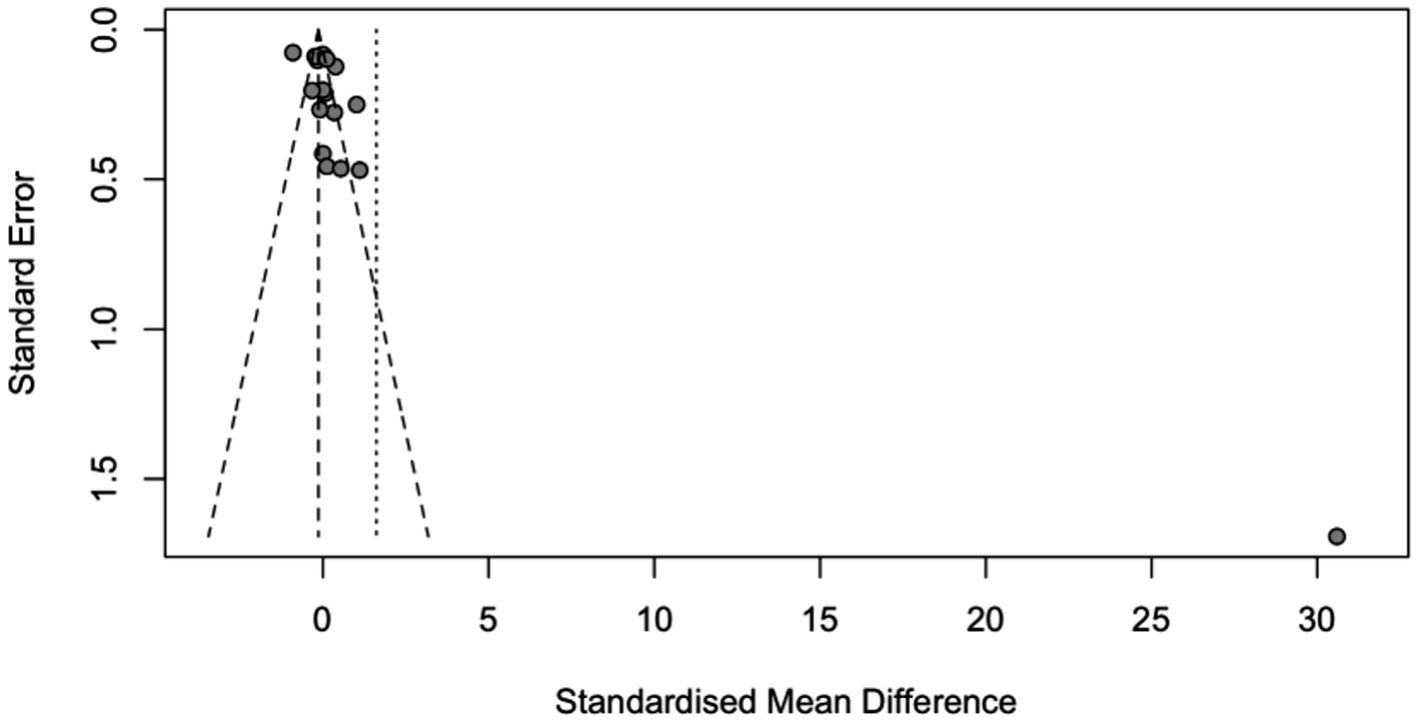
Funnel plot.

When analyzing the funnel plot (Figure 3), the regression test showed that there was asymmetry in the plot (*p* < 0.001). However, the rank correlation test did not yield significant results (*p* = 0.058).

**Figure 4:**
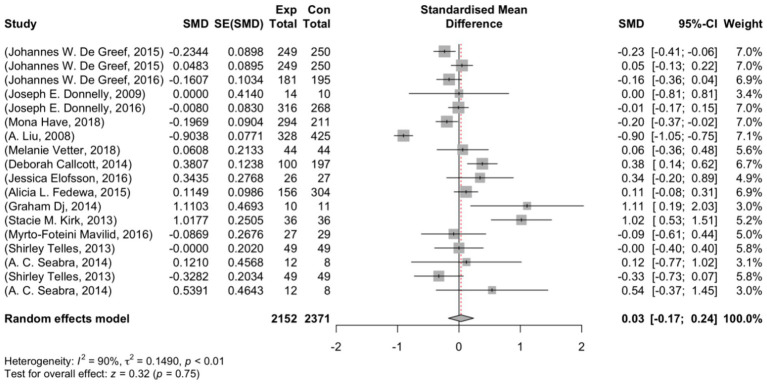
Forest plot showing the observed outcomes and the estimate of the random-effects model.

After removing any potential outlier studies, the analysis was refined to include 18 studies. The outcomes observed ranged from −0.904 to 1.110, and a significant percentage of estimates showed negative effects (44%). By using the random-effects model, the recalibrated estimated average effect size (*μ*) was calculated to be 0.033 (95% CI: −0.172 to 0.239). This aligns with previous findings, as the statistical analysis showed that the average effect size did not differ significantly from zero (z = 0.320, *p* = 0.749). This information is presented in Figure 4, which displays a forest plot comparing the observed outcomes with the estimate obtained from the random-effects model.

**Figure 5:**
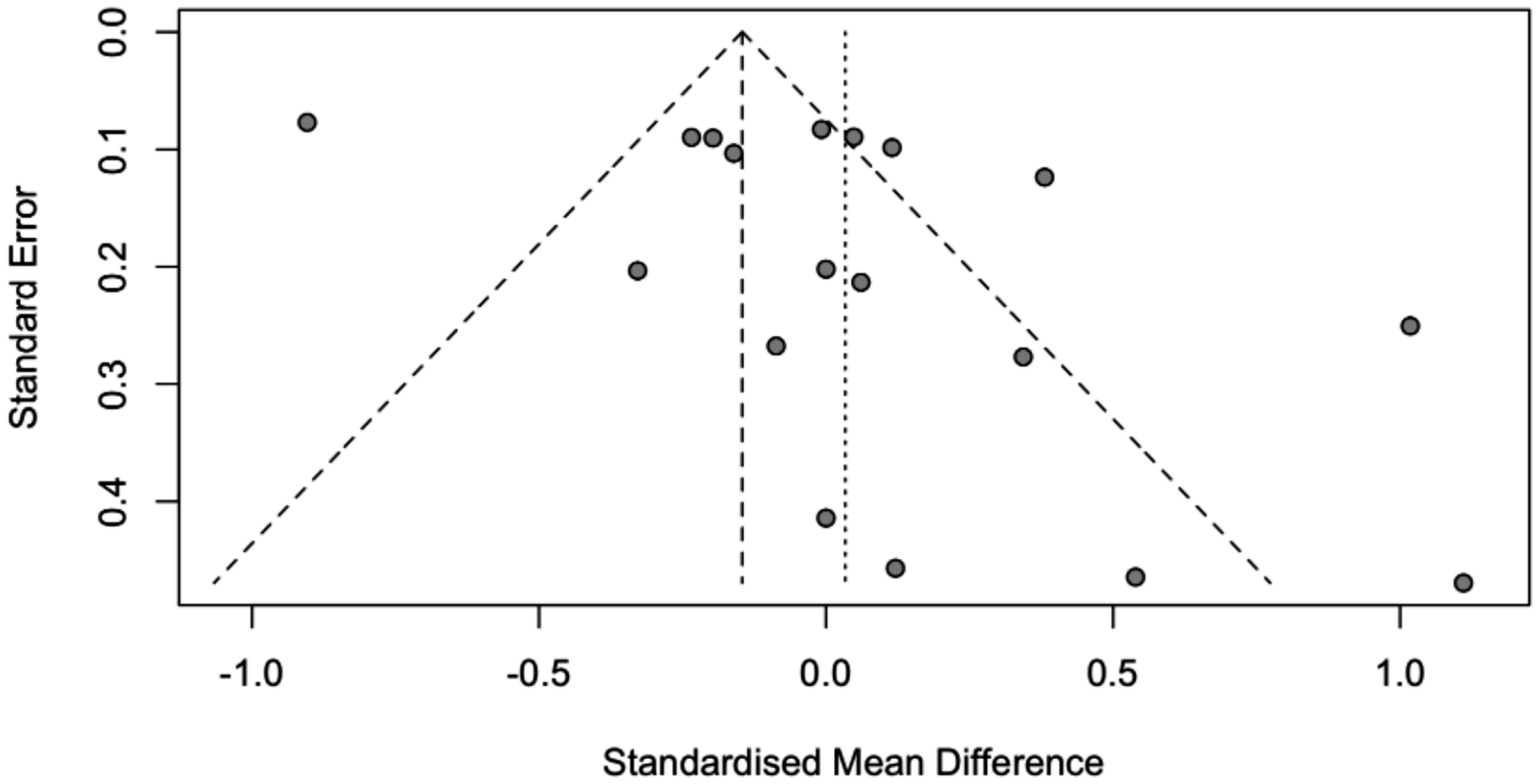
Funnel plot.

After conducting a Q-test analysis, it was found that there is significant variation in the actual results (Q(17) = 167.334, *p* < 0.001, τ^2^ = 0.149, I^2^ = 89.446%). The 95% prediction interval for the true outcomes was calculated to be between −0.750 and 0.817, which means that some studies may have negative results despite the overall positive outcome. Figure 5 shows a visual representation of the estimates. While the rank correlation test did not show any significance (*p* = 0.201), the regression test revealed asymmetry in the funnel plot (*p* = 0.016), indicating a possibility of publication bias.

### Subgroup analysis

**Figure 6:**
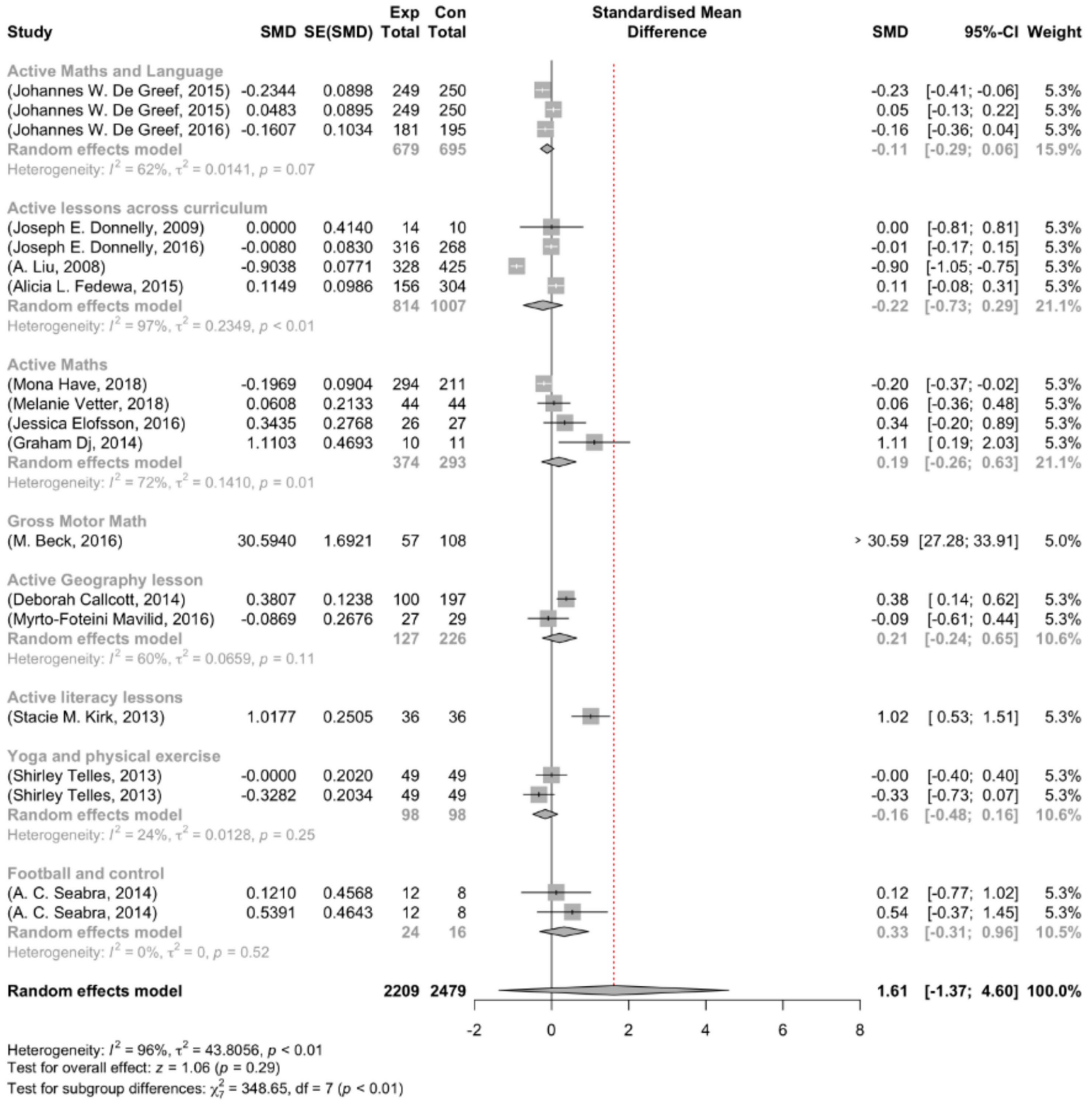
Forest plot of subgrouping analysis.

In Figure 6, you can see the results of the subgroup analysis. It revealed varying effect sizes among the different subgroups. The active math intervention resulted in an SMD in BMI outcomes of −0.1404 (95% CI [−0.3494; 0.0686]) for treatments that focused on physical activity. Similar impact sizes were found for treatments classed as active geography and physical activity, active math, and BMI, with a 0.2071 (95% CI [−0.2357, 0.6499]) and 0.6332 (95% CI [−0.0954, 1.3619]), respectively.

By contrast, interventions denoted as Active Lessons yielded mixed outcomes. One subgroup within this category reported an SMD of −0.5435 (95% CI [−1.4108, 0.3239]), while another subgroup exhibited an SMD of −0.0080 (95% CI [−0.1708, 0.1547]). Similarly, interventions grouped under Active Literacy Lessons displayed an SMD of 0.1149 (95% CI [−0.0782; 0.3081]).

Moreover, interventions focusing on Gross Motor Skills yielded an SMD of [corrected g/SMD] (95% CI [lower, upper]).

Psychological interventions yielded varied outcomes: the subgroup Yoga and Physical Activity showed an SMD of −0.0000 (95% CI [−0.3960; 0.3960]), whereas interventions labeled Football and Cognitive Activities exhibited a more substantial effect size of 0.5391 (95% CI [−0.3709; 1.4492]). Moreover, subgroup analysis indicated significant differences in effect sizes across subgroups (Q = 365.13, df = 14, *p* < 0.0001).

To sum up, the subgroup analysis highlighted the varying effect sizes among different intervention subgroups. Although some subgroups showed significant effects, others had differing levels of impact on the outcomes. Notably, the overall between-group heterogeneity was significant.

(Q = 365.13, *p* < 0.0001), implying a potential divergence in the effects of physical activity interventions across the investigated subgroups.

## Discussion

Our study delves deeper into the multifaceted dynamics between physical education interventions and the holistic well-being of pre-school and school-aged children. While the outcomes did not exhibit statistically significant average effect sizes, they align with prevailing trends from numerous meta-analyses in this domain. These findings reflect the inherent complexities of interventions targeting children, influenced by factors like developmental stages, individual differences, and variability in educational settings shaping nuanced responses (16). Understanding these intricate interplays is crucial in refining our approaches toward tailored and effective physical education strategies for children’s learners.

A key limitation is the lack of statistically significant effects, which could be attributed to the heterogeneity in study populations, intervention designs, and outcome measures included in the meta-analysis. This heterogeneity may have diluted the overall effect sizes. Additionally, there is a potential for publication bias, where studies with null or negative findings are less likely to be published, skewing the evidence base.

A comprehensive comparison of our study’s outcomes with pertinent meta-analyses has significantly augmented our comprehension of the intricate terrain surrounding interventions to enhance children’s well-being. Noteworthy among these insights is the profound realization of the multifaceted nature of intervention outcomes, a point certainly supported by the seminal work of Moreno-Peral et al. (17).

The added layer of complexity when embedding physical activity in educational settings, as noted, is a valid point. However, a conflicting perspective suggests that school-based interventions may have greater real-world applicability and sustainability compared to controlled trials, warranting further investigation into implementation factors.

These contextual nuances resonate deeply with the observations articulated by two other studies (16, 17), illuminating the substantial heterogeneity evident in the effects of physical education interventions on children’s physical and mental health dimensions. Understanding these multifaceted layers becomes pivotal in navigating the intricacies inherent in designing and implementing effective interventions tailored to the unique needs of young learners. This comprehensive understanding not only underscores the complexity but also highlights the need for nuanced, adaptable strategies to optimize the outcomes of such interventions.

A thorough exploration spanning various dimensions is imperative to navigate this field’s intricate web of complexities and propel the collective understanding forward. This involves not only dissecting specific components of intervention design but also amalgamating insights gleaned from previous meta-analyses. Moreno-Peral et al. (17) emphasize the importance of examining participant characteristics to gain insights into how demographic factors like age, gender, and baseline health conditions influence response to interventions. Understanding these nuanced aspects aids in tailoring interventions that align more accurately to the varying needs of individuals.

Moreover, our study champions the use of standardized outcome measures and objective assessments, a sentiment reinforced. The significance of employing such standardized metrics remains paramount in enhancing the comparability of results across various studies. This standardized approach not only ensures consistency but also facilitates a more comprehensive understanding of the efficacy and impact of interventions on children’s well-being. By amalgamating these diverse insights and rigorously addressing these multifaceted elements, we can pave the way for more refined, evidence-based interventions that better serve the nuanced needs of our child population.

Different reviews have been undertaken concerning the child activity profile in the context of effects of physical activity interventions. For instance, Metcalf et al. (10) conducted a systematic review and meta-analysis supportive of a small effect of such interventions on children’s total physical activity levels. Yet, as our results suggest it is very important to put these findings in a broader context of activities which summarize the child daily routine.

The relatively small effect size regarding the change in overall physical activity level suggests that interventions need to be placed sensitively within the complex weave of children’s activity. There is a need to understand better the interactions between interventions and the diverse activities of children. Such an understanding forms a basis for the design of more tailored and effective approaches toward optimization of the impact of interventions aimed at fostering healthier and more active lifestyles among children (10).

There has been a lot of research on the impact of physical activity interventions on children’s overall patterns of activity. The most recent systematic review and meta-analysis by Metcalf et al. indicated that there was a limited impact on total activity levels. The findings of the current study support these observations but this should be placed into context in terms of the whole activity lives of children.

The realm of educational interventions aimed at enhancing health-related physical fitness outcomes has gained significant attention. García-Hermoso et al. (18) emphasizes the importance of optimizing the quality and quantity of physical education programs to improve fitness outcomes. However, their analysis may be limited by the heterogeneity in intervention designs and outcome measures across included studies (18, 19).

To address this limitation, our study advocates for the use of standardized outcome measures and objective assessments, as reinforced by Simonton et al. (16). This approach enhances the comparability of results across studies and provides a more comprehensive understanding of the efficacy and impact of physical education interventions on children’s physical fitness and overall well-being.

By acknowledging these potential limitations and biases, we can refine our understanding of how physical education interventions influence children’s overall activity patterns and physical fitness. Exploring compensatory behaviors, utilizing objective measures, and employing standardized outcome assessments can lead to more accurate and nuanced insights to guide the development of effective interventions tailored to children’s needs.

Furthermore, the compelling positive impacts arising from physical education interventions on mental health outcomes have unveiled a transformative narrative in the current study. Cho’s (3) comprehensive research, which is supported by our study, sheds a striking light on the impact of physical education in fostering emotional competence and reducing symptoms of anxiety and depression. These profound findings not only spotlight the integral role of physical education but also echo its significance in the broader spectrum of fostering holistic well-being among children (10). It is clear from the results of these studies that physical education transcends the realms of mere physical effort, playing an important role in bolstering mental resilience and emotional wellbeing in children.

Furthermore, physical education interventions should address the escalating obesity rates. A number of observational studies have consistently demonstrated a symbiotic relationship between heightened physical activity levels, weight loss, and lower obesity risks (10). These aligned narratives resonate deeply with the focal point of our study, which accentuates the multifaceted and comprehensive health implications inherent in physical education interventions. In addition to targeting physical health, these interventions are highly effective tools in combating numerous health challenges, positioning themselves as pivotal components in addressing the rapidly expanding obesity epidemic.

In this study, we combine findings from various meta-analyses to illustrate the intricate tapestry of dynamics which underlie the relationship between physical education interventions and children’s well-being in general. Acknowledging the inherent shared challenges and complexities within this sphere, it becomes increasingly evident that embracing a comprehensive and multifaceted approach is imperative for charting the course of future research endeavors in this domain. Moreover, it is crucial to recognize the potential limitations within our study, such as the prospect of publication bias, variations in study heterogeneity, and the quality of available evidence. These acknowledgments serve as guideposts, offering valuable insights for refining future research trajectories and delineating the parameters for a more profound exploration of physical education’s impact on children’s holistic health and well-being.

### Future study

In future studies, it is essential to provide clearer reporting on implementation details. Additionally, researchers should describe the activities of the control group and specify whether the intervention adds to or replaces ordinary physical education lessons. This transparency will facilitate the interpretation of results and address limitations. Researchers should also explore alternative explanations for the observed outcomes by incorporating a broader range of outcome measures and considering factors such as the choice of measurement tools and specific metrics used, which could have influenced the results.

### Strengths and limitations

The review exhibits several strengths. Firstly, it includes a comprehensive literature search across four high-quality databases. Additionally, the search process, data extraction, and quality assessment were conducted independently by two researchers. Furthermore, the study selection process prioritized studies where parental or other interventions were not implemented, while also considering studies which had similarities in physical and mental health outcomes.

The review also acknowledges certain limitations. Despite searching across four databases, there is a possibility of missing relevant articles in languages other than English. Additionally, the interventions exhibited significant variation, as did the control groups, leading to considerable heterogeneity in effect sizes. Furthermore, the study excluded research with differing physical and psychological outcomes.

## Conclusion

Overall, evidence from randomized trials suggests that physical education interventions may improve certain outcomes. However, pooled effects were heterogeneous and statistically uncertain. More rigorous, standardized trials are needed to draw definitive conclusions. Future research should continue to explore the optimal strategies and components of physical education interventions to maximize their impact on the health and well-being of children and adolescents.

## Data Availability

The raw data supporting the conclusions of this article will be made available by the authors, without undue reservation.
